# Diversity of Algerian oases date palm (*Phoenix dactylifera* L., Arecaceae): Heterozygote excess and cryptic structure suggest farmer management had a major impact on diversity

**DOI:** 10.1371/journal.pone.0175232

**Published:** 2017-04-14

**Authors:** Souhila Moussouni, Jean-Christophe Pintaud, Yves Vigouroux, Nadia Bouguedoura

**Affiliations:** 1 Université des Science et de la Technologie Houari Boumediene (USTHB), Faculté des Sciences Biologiques, Laboratoire de Recherche sur les Zones Arides (LRZA), Alger, Algeria; 2 Institut de Recherche pour le Développement, UMR DIADE, DYNADIV, Montpellier, France; National Cheng Kung University, TAIWAN

## Abstract

Date palm (*Phoenix dactylifera*L.) is the mainstay of oasis agriculture in the Saharan region. It is cultivated in a large part of the Mediterranean coastal area of the Sahara and in most isolated oases in the Algerian desert. We sampled 10 oases in Algeria to understand the structure of date palm diversity from the coastal area to a very isolated desert location. We used 18 microsatellite markers and a chloroplast minisatellite to characterize 414 individual palm trees corresponding to 114 named varieties. We found a significant negative inbreeding coefficient, suggesting active farmer selection for heterozygous individuals. Three distinct genetic clusters were identified, a ubiquitous set of varieties found across the different oases, and two clusters, one of which was specific to the northern area, and the other to the drier southern area of the Algerian Sahara. The ubiquitous cluster presented very striking chloroplast diversity, signing the frequency of haplotypes found in Saudi Arabia, the most eastern part of the date palm range. Exchanges of Middle Eastern and Algerian date palms are known to have occurred and could have led to the introduction of this particular chlorotype. However, Algerian nuclear diversity was not of eastern origin. Our study strongly suggests that the peculiar chloroplastic diversity of date palm is maintained by farmers and could originate from date palms introduced from the Middle East a long time ago, which since then, hasbeen strongly introgressed. This study illustrates the complex structure of date palm diversity in Algerian oases and the role of farmers in shaping such cryptic diversity.

## Introduction

The date palm (*Phoenix dactylifera* L., Arecaceae) is a perennial monocotyledon (2n = 36). It is an ecologically, culturally and economically important crop, widely cultivated in arid and semi-arid Mediterranean regions, in the Sahara, and in the Middle East [1, 2, 3]. More than 3,000 cultivars are estimated to be used for date production worldwide, of which around 60 are widely grown and have important national and international markets [4].

Early cultivation of the date palm is recorded in the eastern part of its cultivation area, in southern Mesopotamia in the 5^th^ Millennium BC. In the western part of its cultivation area, evidence for domesticated date palm has been found in Egypt in the 4^th^ century BC [5]. Genetic studies of date palm clearly separate the eastern and western group [6, 7, 8, 9, 10]. The two primary gene pools (eastern and western) are also observed in the maternally-inherited chloroplast genome [7] and the paternally-inherited Y chromosome [11]. Two major alleles were found in the chloroplast, an occidental and oriental haplotype [7].

In Algeria, chloroplast diversity includes roughly 70% of the eastern chloroplast [10]. In neighboring Egypt, Tunisia and Morocco, the proportion of eastern haplotype only ranges from11% to 42%, but Algerian nuclear diversity is similar to that found in its neighboring countries, Tunisia and Morocco [10]. The contrast between nuclear and chloroplast diversity observed in Algeria thus remains largely unexplained, perhaps because of the only fragmentary analysis of the diversity of the 1,000 varieties described in Algeria.

Date palms are cultivated in Algerian oases in most of the regions south of the Saharan Atlas Mountains. In 2002, Algerian date palm groves contained 13.5 million trees occupying 120,830 ha, whilein 2015, 18 million date palms occupied 169,380 ha[12]. Nearly 1,000 cultivars clonally propagated from offshoots have been inventoried and their distribution shows a very marked breakdown into eastern, central and western parts of the country. Some cultivars are found in two or three regions but most are restricted to their area of origin [13].What is more, cultivars are not evenly distributed across oases, as they are adapted to slightly different types of soil, ranges of temperature and humidity, and often do not produce a satisfactory yield when cultivated outside their place of origin [14, 12]. Palms grown from seeds occur randomly in the oases and are called “khalts” or “dgouls”. Khalts represent up to 10% of a population of date palm sand area valuable resource for new selection by farmers.

The aims of the present study were to (1) genetically characterize date palm agrobiodiversity in Algeria using a set of representative female fruiting date palm cultivars and (2) investigate the structure of genetic diversity in the oases south of the Saharan Atlas based on nuclear and chloroplast genotyping.

## Materials and methods

### Ethics statement

No specific permissions are required for the activities conducted in this study. The study did not involve endangered or protected species.

### Plant material, DNA extraction and quantification

**Fig 1:**
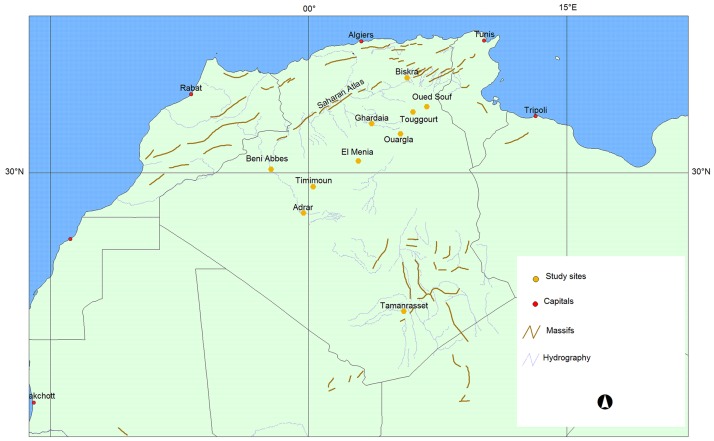
Map showing the distribution of the 10 oases sampled in Algeria. The 414 samples came from the following sources: Biskra 13 cultivars; Oued Souf 22 cultivars; Touggourt 30 cultivars; Ouargla 5 cultivars; Ghardaia 17 cultivars; El Menia 10 cultivars; Timimoun 16 cultivars; Adrar 18 cultivars; Beni Abbes 14 cultivars, and Tamanrasset 4 cultivars.

A total of 414 samples of date palm (*Phoenix dactylifera* L.) were sampled from 10 oases distributed in the north-western (3 oases), southern (1 oases), north-central (2 oases) and north-eastern (4 oases) date palm regions of Algeria (Fig 1). The plant material consisted in portions of young leaflets collected from 12–15 year-old palms whenever possible. Fresh material was dried in silica gel and stored at room temperature. The 414 samples corresponded to 114 named female varieties, 14 unidentified female varieties and 10 male plants. DNA was extracted from 45 mg of dried leaf tissue. The samples were ground to fine powder in a TissueLyser II homogenizer (Qiagen), in the presence of steel balls. DNA was extracted using the DNeasy Plant MiniKit (Qiagen) following the manufacturer’s instructions. DNA quality was checked by electrophoresis on 1% agarose gel [15]. The DNA concentration was determined using a NanoDrop spectro-photometer.

### SSR genotyping

One dodecanucleotide plastid minisatellite located in the *trnG-trnfM* inter gene space was genotyped [7]. The occidental chlorotype is characterized by three repetitions of the 12 bp motif (type 3 chlorotype), while the oriental chlorotype has four repetitions (type 4 chlorotype) [16, 17]. This polymorphism provides the easiest access to the differentiation of the occidental and oriental chloroplast genomes, which is otherwise extensive. Indeed, sequence alignment of ca. 50 kb of an occidental chloroplast from Elche, Spain and an oriental one (cv Khalass) from the Saudi Arabian peninsula, revealed 18 SNPs along with a number of other mutations, including microsatellites, indels and small inversions [18]. The comparison of two complete sequences of oriental chloroplast genomes of cv Khalass and Aseel [19] resulted in only 3 SNPs. The two amplified alleles at this locus are 242 bp and 254 bp in length.

The following reaction mixture was used for genotyping with *trnG-trnfM*-primers: 1 μl of DNA, PCR buffer, 5 μl of FailSaif premix, 0.1 μl of Taq DNA polymerase, 0.1 μl forward primer, 0.1 μl of antisense primer, and water qs 3.6 μl Promega to reach a final volume of 10 μl. The amplification protocol comprised a denaturation step at 95°C for 3 minutes followed by 30 cycles each comprising a denaturation step at 94°C for 30 seconds, an annealing step at 56°C for 1 minute 30 seconds, and an elongation step at 72°C for 1 minute 30 seconds. Genotyping was performed with the Qiaxel DNA analyzer.

Eighteen nuclear microsatellite loci were also used for this study (Table 1). These loci were analyzed on 192 samples including 178 female clones representing 114 identified varieties, 11 unidentified female clones, one seed-grown female genotype and 2 male plants. The individuals sampled were collected from the following locations: Biskra (13 cultivars / 20 individuals), Oued Souf (22 cultivars / 30 individuals), Touggourt (30 cultivars / 32 individuals), Ouargla (5 cultivars / 5 individuals), Ghardaia (17 cultivars / 29 individuals), El Menia (10 cultivars / 17 individuals), Timimoun (16 cultivars / 17 individuals), Adrar (18 cultivars / 20 individuals), Beni Abbes (14 cultivars / 15 individuals) and Tamanrasset (4 cultivars / 7 individuals) (S1 Table).

**Table 1:** Summary data for 18 microsatellite loci.

Marker name	Short name	Forward primer sequence (5’→ 3’)	Reverse primer sequence (5’→ 3’)	SSR motifs	Length (pd)	At (C°)	Reference	GeneBank accessions
mPdCIR010	Pd10	ACCCCGGACGTGAGGTG	CGTCGATCTCCTCCTTTGT	(GA)_22_	164	56	[20]	AJ571673
mPdCIR015	Pd15	AGCTGGCTCCTCCCTTCTTA	GCTCGGTTGGACTTGTTCT	(GA)_15_	135	52	[20]	AJ571674
mPdCIR016	Pd16	AGCGGGAAATGAAAAGGTAT	ATGAAAACGTGCCAAATGTC	(GA)_14_	134	52	[20]	AJ571675
mPdCIR025	Pd25	GCACGAGAAGGCTTATAGT	CCCCTCATTAGGATTCTAC	(GA)_22_	230	49	[20]	AJ571676
mPdCIR032	Pd32	CAAATCTTTGCCGTGAG	GGTGTGGAGTAATCATGTAGTAG	(GA)_19_	300	52	[20]	AJ571677
mPdCIR035	Pd35	ACAAACGGCGATGGGATTAC	CCGCAGCTCACCTCTTCTAT	(GA)_15_	188	54	[20]	AJ571678
mPdCIR057	Pd57	AAGCAGCAGCCCTTCCGTAG	GTTCTCACTCGCCCAAAAATAC	(GA)_20_	282	55	[20]	AJ571682
mPdCIR063	Pd63	CTTTTATGTGGTCTGAGAGA	TCTCTGATCTTGGGTTCTGT	(GA)_17_	167	50	[20]	AJ571683
mPdCIR078	Pd78	TGGATTTCCATTGTGAG	CCCGAAGAGACGCTATT	(GA)_13_	121	50	[20]	AJ571685
mPdCIR085	Pd85	GAGAGAGGGTGGTGTTATT	TTCATCCAGAACCACAGTA	(GA)_29_	179	50	[20]	AJ571686
PdAG1-ssr	AG1	TCTGATTTCGTTTACTTCTTAGGA	TTCATATTCAGTTGTCGGGTGTA	(GA)	260	52	[21]	
PdCUC3-ssr1	CUC3-1	CGTGGACTCATGACTCGCATGTCC	GGTCCTTGCCGGTGGCCTTC	(GT)_14_	330	60	[22]	HM622273
PdCUC3-ssr2	CUC3-2	ACATTGCTCTTTTGCCATGGGCT	CGAGCAGGTGGGGTTCGGGT	(GA)_22_	350	59	[22]	HM622273
PdAP3-ssr	AP3	GAGAAATAGAGAGCTGTGCAAG	CTGCAGTACTCGGAGAACTTG	(GA)_25_	331	57	[23]	KC188337
mPdIRD013	P13	GCGGAGACAGGAGATGGTAA	CTTGACTGCTTCTGCTGCTG	(CAC)_6_	204	60	[24]	PDK_20s1496731g002
mPdIRD031	P31	GCAGGTGGACTGCAAAATCT	CTATTGGGGTGCTGATCCAT	(CCA)_7_	198	60	[24]	PDK_20s1419261g003
mPdIRD033	P33	GGAGCATACAGTGGGTTTGC	CAGCCTGGGAATGAGGATAG	(CAG)_7_	199	60	[24]	PDK_20s1569281g001
mPdIRD040	P40	GAGAGATGCGTCAGGGAATC	CCAGAATCTTCCAAGCAAGC	(CCAGTG)_4_	193	60	60	PDK_20s1327401g002

Information concerning the microsatellite markers used to genotype date palm cultivars (identified from the genome of *Phoenix dactilyfera)* using the primer sequences. The SSR motifs, PCR product size, primer annealing temperatures, referenced and GeneBank accession numbers are given. PCR = polymerase chain reaction; SSR = simple sequence repeat; At = annealing temperature.

For all DNA extracts, the concentration was homogenized at 5 ng/μl. Amplification reactions were performed in a final volume of 20 μl containing 15 ng of template DNA, 10× reaction buffer, 5 pmol each of forward and reverse primer, 0.2 mM of each deoxynucleotide, 2 mM MgCl_2_, and 1 unit of Taq polymerase (Sigma). The forward primers were 5’ labelled with one of three fluorescent compounds (6-FAM, NED or HEX) to enable analysis with automated sequencers. PCR was carried out using an Eppendorf Mastercycler pro equipped with vapor protect technology (AG, Hamburg, Germany). The PCR conditions were: an initial denaturation step at 95°C for 5 min, followed by 35 cycles each consisting of denaturation at 95°C for 30 seconds, hybridization at 51–57°C for 60 seconds and elongation at 72°C for 30 seconds, and a final step at 60°C for 30 min. Amplified products were run on an ABI 3130XL Genetic Analyzer (Applied Biosystems, USA) and alleles were scored using the GeneMapper V3.7 software (Applied Biosystems).

### Diversity and statistical analysis

Genetic diversity parameters, i.e. the number of alleles per locus (Na), allelic frequencies, heterozygosity and polymorphism information content value (PIC value) were estimated with Power Marker version 3.25 software [25].

F_IS_ and F_ST_ indices were calculated according to Weir and Cockram [26] using Fstat [27] and Genetix 4.05 software [28]. Allelic richness was computed with Fstat [27].

Anon-parametric Wilcoxon test was used to determine the significance of differences in diversity between groups using R [29]. Probability values (P-value) of the test are given for each comparison between pairs of populations.

Cluster analysis was conducted by generating a genetic distance matrix by calculating the shared allele frequencies using the neighbor-joining algorithm implemented in Mega version 5.05 software [30]. We also assessed clustering based on the Bayesian approach implemented in STRUCTURE version 2.3 [31]. We assessed different numbers of groups (K) ranging from 1 to 10, with 10 repetitions for each given value of K. For each run, we used a burn in period of 10,000 iterations, and a post burn in simulation length of 1,000,000. The most probable number of clusters was assessed using both the likelihood and an *ad hoc* quantity based on the second order rate of change in the log probability of data between different **K** values [32]. Diversity (number of alleles, heterozygosity) was assessed in the different clusters and compared using the Wilcoxon test. We assigned an individual to a group if its ancestry in this group was > 75% [33]. To check for a difference in chlorotype frequency between groups, we performed a Chi-square test, using SPSS Statistics 20.

## Results

### Chloroplast minisatellite and nuclear SSR genotyping

**Fig 2:**
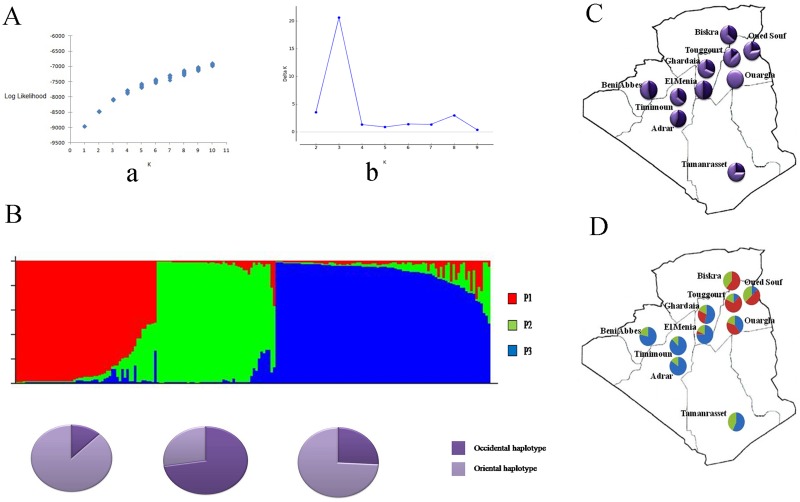
Bayesian cluster analysis using the STRUCTURE program: Results for K = 3. (A) Plots of (a) maximum log likelihood over the 10 runs and (b) delta k from the structure analysis was calculated according to the method of Evanno et al. (B) Estimated population structure inferred from all the individual date palms for K = 3. Each individual is represented by a thin vertical line divided into K colored segments representing the fraction of the individual’s estimated membership of the K clusters. Pie charts show the frequencies of the haplotypes belonging to the three structure groups. (C) Geographic distributions of the 10 oases and two haplotypes. The pie chart shows the proportions of haplotypes in each oasis. (D) Sampling location of the date palms. Pie charts show the proportion of membership of each sampled population inferred by structure analysis (K = 3).

The two genotyping methods used (either Qiaxel or the ABI genetic analyzer) were consistent and revealed two chlorotypes, previously reported as occidental and oriental [7] (S2 Table). The occidental (western) chlorotype was found in 31.3% and the oriental (eastern) in 68.7% of the total sample. The proportion varied among oases (Fig 2C).

Linkage disequilibrium was very low between SSR markers (S3 Table). A total of 143 alleles were identified with the 18 nuclear markers. The average number of alleles was 7.9, average expected heterozygosity was 0.60, and the average PIC was 0.57 (Table 2). The average number of alleles per locus at the individual oases ranged from 3.83 (Ouargla) to 5.78 (Oued Souf). Allelic richness varied slightly between oases with values ranging from 3.36 (Touggourt) to 3.85 (Beni Abbes). The same was true of expected heterozygosity, which ranged from 0.53 (Ouargla) to 0.59 (Oued Souf and Beni Abbes), and of observed heterozygosity, which ranged from 0.56 (Tamanrasset) to 0.65 (Ghardaia).

**Table 2:** Descriptive genetic parameters for 18 microsatellite loci analyzed on 192individual date palms.

Marker	N° of genotypes	N° of alleles	Major allele frequency	Gene diversity	Expected heterozygosity He	PIC
mPdCIR010	31	10	0.28	0.80	0.86	0.78
mPdCIR015	19	8	0.50	0.70	0.68	0.67
mPdCIR016	8	4	0.51	0.61	0.56	0.53
mPdCIR025	19	10	0.29	0.77	0.87	0.74
mPdCIR032	25	9	0.41	0.75	0.77	0.72
mPdCIR035	15	7	0.70	0.48	0.27	0.46
mPdCIR057	18	7	0.40	0.72	0.77	0.68
mPdCIR063	14	6	0.34	0.75	0.74	0.71
mPdCIR078	41	16	0.31	0.84	0.83	0.82
mPdCIR085	30	10	0.25	0.83	0.84	0.80
PdAG1-ssr	54	19	0.27	0.87	0.86	0.86
PdCUC3-ssr1	1	1	1.00	0.00	0.00	0.00
PdCUC3-ssr2	42	12	0.17	0.88	0.85	0.87
PdAP3-ssr	20	9	0.37	0.74	0.83	0.70
mPdIRD013	2	2	0.98	0.05	0.05	0.05
mPdIRD031	5	3	0.83	0.29	0.31	0.26
mPdIRD033	5	4	0.91	0.16	0.16	0.16
mPdIRD040	9	5	0.70	0.47	0.49	0.43
Mean	19.89	7.94	0.51	0.59	0.60	0.57

The number of genotypes, the number of alleles, the frequency of the main alleles, gene diversity, heterozygosity and the polymorphism information content (PIC) of 18 loci in *Phoenix dactyifera* are given.

PIC = polymorphism information content.

No significant difference in diversity (allelic richness, heterozygosity observed or expected) between oases was observed using a Bonferroni corrected p-value for multiple testing (S4, S5, S6 Tables). Significant negative fixation indices (F_IS_) were obtained for two out of 10 oases (Table 3). Only three isolated southern oases: Tamanrasset, Adrar and Beni Abbes, had a deficit of heterozygous individuals (Table 3).

**Table 3:** Summary statistics for 18 microsatellite loci in 10 oases.

Oasis	N°	Na	Ar	Ho	He	F_IS_	P value F_IS_
Biskra	18	4.72	3.45	0.58	0.56	-0.02	0.3216
Touggourt	32	5.33	3.36	0.59	0.54	-0.06	0.0097
Oued Souf	30	5.78	3.73	0.60	0.59	-0.01	0.2457
Ouargla	5	3.83	3.83	0.61	0.53	-0.05	0.2563
Ghardaia	29	5.22	3.53	0.65	0.56	-0.13	0.0000
Tamanrasset	7	4.11	3.68	0.56	0.57	0.09	0.9603
El Menia	17	4.78	3.47	0.58	0.55	-0.03	0.1719
Timimoun	16	5.11	3.67	0.64	0.57	-0.04	0.1694
Adrar	20	5.06	3.53	0.58	0.56	0.004	0.5505
Beni Abbes	14	5.22	3.85	0.58	0.59	0.05	0.9013

The number of samples (N°), the number of alleles per locus (Na), allelic richness (Ar), observed heterozygosity (Ho), expected heterozygosity (He), theinbreeding coefficient (F_IS_) and the p-value of F_IS_ are given for each population (averaged across the 18 loci).

### Genetic differentiation between populations

Overall differentiation was weak between oases, with an overall average differentiation of 0.0119. Overall, there was isolation by geographical distance (Mantel test, p-value = 0.039). Geographically close populations were generally weakly differentiated while greater differentiation was observed for populations sampled further apart (Table 4).

**Table 4:** Genetic distance between oases (F_ST_).

	Biskra	Touggourt	Oued Souf	Ouargla	Ghardaia	Tamanrasset	El Menia	Timimoun	Adrar	Beni Abbes
Biskra		0.0288*	0.0288	0.0062	0.0701*	0.0205	0.0668*	0.0581*	0.0559*	0.0623*
Touggourt			0.0098	-0.0288	0.0379*	0.0473*	0.0420*	0.0479*	0.0492*	0.0555*
Oued Souf				-0.0065	0.0240*	0.0270	0.0289	0.0322*	0.0334*	0.0313*
Ouargla					0.0197	0.0049	0.0016	0.0063	0.0079	0.0132
Ghardaia						0.0322	0.0414*	0.0143	0.0365*	0.0372*
Tamanrasset							0.0245	0.0141	0.0129	0.0172
El Menia								0.0179	0.0072	0.0250
Timimoun									0.0119	0.0056
Adrar										0.0086
Beni Abbes										

The F_ST_ component was determined using a method that calculates the genetic distance between two oases, the differences being in pairs. A Bonferroni correction was applied, the significance level passing p < 0.001.

* P < 0.001 (significant).

### Variety name and diversity

Based on an individual phylogenetic tree, relationships between varieties with similar names are easy to see. Varietal names need to be compared because of the many synonyms, such as: Al Kayed and Tanteboucht; Tgaza and Takerboucht; Halimi and Masri; Aharthan and Harthan Oumazer; Chikh, Mhammed Chikh and Hamuri; Bent Cherk and Cherka; Tati and Tacherwint; Kesba and Sokrya; Azizaou and Adam Zrak; Alig, Bu’Rus and Our’Rous. In a few cases, we also observed varieties that shared the same name but were relatively distant. For example, three samples of the variety Hamraya collected at three different oases (Biskra, Touggourt and Oued Souf) differed genetically.

### Population structure and differentiation

Analysis of population structure led us to keep three sub-populations (Fig 2) based on Evanno statistics (Fig 2b). Among the 192 accessions with distinct genotypes, 186 were assigned to a single group (Fig 2). Only six accessions remained "unclassified" and were considered "admixed". The first group included 51 varieties from the north-eastern and north-central part of the Algerian Sahara. The second group, which was the smallest with 48 varieties, was present in every oasis. The third group was the largest with 87 varieties, including varieties from the north-western, north-central and southern part of the Algerian Sahara, and 20% from the north-eastern part.

Allelic richness was lowest in the northern group (Table 5). No difference in observed and expected heterozygosity was found between the three groups (Table 5, Wilcoxon signed-rank test, p-value> 0.05).The inbreeding coefficient F_IS_ was negative and significant in the first and third group (Table 5). Only group 2 had no excess of heterozygotes. Pairwise F_ST_ values in the three groups ranged from 0.066 to 0.092 (Table 6). The highest differentiation was found between group 1 and group 2 (F_ST_ = 0.0924). The three groups are thus genetically different (significantly different at p < 1%).

**Table 5:** Description and variability parameters of the 18 polymorphic microsatellite loci in the three STRUCTURE populations.

Population	N°	Na	Ar	Ho	He	F_IS_	P value F_IS_
Population 1	51	5.28	5.15	0.60	0.53	-0.13	0.000002
Population 2	48	6	5.88	0.57	0.56	-0.004	0.426301
Population 3	87	6.94	6.32	0.61	0.58	-0.05	0.000101

The number of samples (N°), the number of alleles per locus (Na), allelic richness (Ar), expected heterozygosity (He), observed heterozygosity (Ho), the inbreeding coefficient (F_IS_) and the p-value of F_IS_ are given for each population and each locus.

**Table 6:** Genetic distance between Structure populations (F_ST_).

	population 1	population 2	population 3
population 1		0.0924*	0.0742*
population 2			0.0659*
population 3			

The F_ST_ component was determined using a method that calculates the genetic distance between two assumed populations, the differences being in pairs. A Bonferroni correction was applied, the significance level passing p < 0.01.

* p < 0.01 (significant)

A relationship was found between the three genetic structure clusters and the two chlorotype frequencies (Fig 2, χ^2^ = 28.1, dof = 2, p<0.001).

### Genetic distance and dendrogram construction

**Fig 3:**
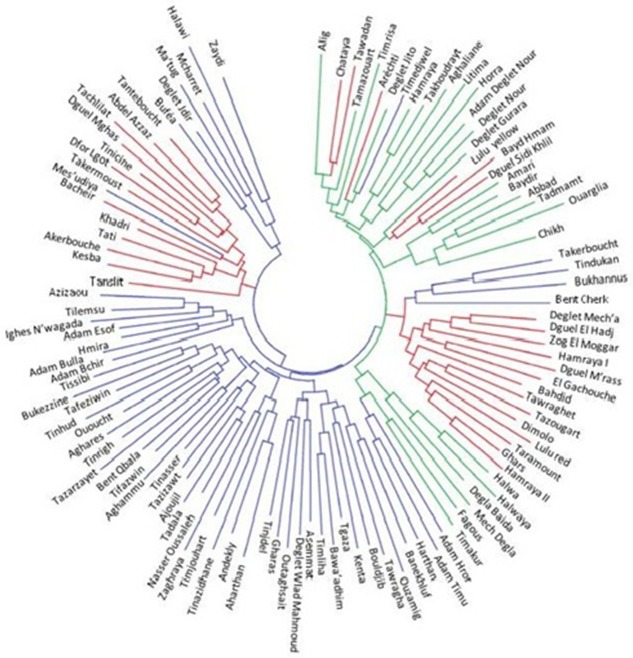
Neighbor-joining tree with microsatellite genotypes using shared allele distance. The neighbor-joining tree shows the genetic relationships among the date palm genotypes included in the study, where each branch represents a single individual. The individuals in the populationSTRUCTURE1are in red, those in population 2 in green, and those in population 3 in blue.

The matrix of estimated distances between individuals of the total population was used to construct a phylogenetic tree of individuals in the total population. The distances ranged from 0 to 0.75, underlining the wide genetic variability of our study population. Zero distance between two individuals suggests a clonal relationship (Fig 3).

Analysis of the phylogenetic tree showed that the individuals were grouped independent of their geographic origin and ethnic name ecotype. The distribution on the phylogenetic tree can be explained by the existence of a common genetic basis between different populations and ecotypes, despite geographic distance and phenotypic divergence.

## Discussion

### Genetic diversity and population structure of date palm

The diversity of Algerian date palm was shown to be high with an average number of alleles per locus of 7.94, similar to the number observed in Qatar, Iraq or Tunisia [34, 35, 36, 37, 38]. Seven populations had negative fixation indices (F_IS_), suggesting an excess of heterozygosity at these loci. Two populations, Touggourt (F_IS_ = -0.06) and Ghardaia (F_IS_ = -0.13), had significant positive fixation indices (F_IS_). A negative F_IS_ value suggests that different heterozygous genotypes have been maintained. Negative and significant F_IS_ value were also found when individuals were grouped based on Bayesian analysis of population structure. This particular signature is not very common in plants, and may indicate direct selection for individuals with the best performance, i.e. those with high heterozygosity. It suggests farmers had a direct impact on maintaining the most heterozygous plants [39]. As date palms are also propagated by stem (offshoots), it should be noted that if all the plants were at Hardy-Weinberg equilibrium, propagation by stem would not change or increase F_IS_ through the production of clones. So this excess of heterozygous plants could only have been be maintained by choosing the fittest individual [39].

The difference in F_ST_ between oases was relatively low, ranging from 0.0016 to 0.0701. However, slightly higher differentiation was observed between oases in the northern and southern Algerian Sahara. This could reflect the use of different varieties in different ecological conditions. In the Algerian Sahara, the climate is characterized by aridity and heat, and gradually becomes hotter and drier from north to south. The northern regions of the Sahara are characterized by a semi-arid climate, which is the case of Biskra located at the foot of the Saharan Atlas mountain range, the natural boundary between northern and southern Algeria. Similarly, Touggourt, Oued Souf, Ouargla and Ghardaia havea semi-arid to arid climate. Southern Saharan regions, i.e. Beni Abbes, Adrar, Timimoun and Tamanrasset are characterized by an extremely hot dry desert climate. The analyses of structure also support the northern and southern grouping of diversity.

### Explanation for the genetic history of diffusion of date palm cultivars in Algeria

Recent studies based on the global diversity of date palm suggest a geographic differentiation between African and Middle Eastern date palms [10, 8]. Chloroplastic diversity and Pairwise the non-recombinant portion of the segment of the Y chromosome both reveal differentiation between African and Middle Eastern cultivars [11]. A hypothesis of two origins, one in the east and the other in the west has been proposed [10, 8]. However, it is likely that this differentiation was caused by successive bottlenecks during the diffusion of cultivated date palms. However that may be, the diversity of the chloroplast is structured with major eastern and western alleles. Chloroplast genomes help trace the path of the female lines [7], and our chloroplast data provided insight into this maternal structure.

Interestingly, we observed a high frequency (almost 75%) of the eastern (oriental) chloroplast in a specific group of varieties. In this particular group, the frequency was similar to that found further east. Conversely, in the two other structure groups, chloroplastic allele frequency was similar to that observed further west.

The 18 microsatellite loci used in the study differed between eastern and western date palms [10]. Algeria does not differ particularly from other western countries and falls in the western genetic group. While chloroplasts inherited maternally suggest some proximity to the east, the nuclear markers clearly show a proximity to the west. One hypothesis proposed to explain this peculiar pattern, is that seeds and offshoots were imported from the Middle East to North Africa, and, after generations of crossing with local males (western nuclear genome), varieties acquired a western nuclear genome although an eastern chlorotype was maintained. Such varieties therefore carry the eastern haplotype. Another possible explanation is that both eastern and western haplotypes were widespread in both the eastern and western Sahara, but because farmers selected a specific genetic group of plants, chloroplastic diversity varied considerably from one local genetic group to the other in the west. Whatever the scenario, this pattern is only compatible with human selection that maintained the maternal lineage separately. The fact that we also found that in Algeria, nuclear SSR is structured in three groups shows that the groups were also to a certain extent kept separate by controlled pollen flow.

In conclusion, humans shaped the diversity of date palms by selecting heterozygous individuals and maintaining them. Moreover, cryptic diversity was observed both at genome and chloroplast level. Taken together, our results suggest that human selection played a major role in maintaining the diversity and lineage of date palms in Algeria.

## Supporting information

S1 Fig Clustering of individuals by STRUCTURE at K = 2 and K = 3. Individuals are represented by vertical colored lines. Individuals of the same color belong to the same cluster. Individuals with several different colors show the percentage of the genome that was inherited from each cluster.
(TIF)

S1 Table List of samples used in nuclear SSR genotyping. (PDF)

S2 Table Genetic assignment of the sample cultivars according to chlorotype and STRUCTURE grouping for K = 3. (PDF)

S3 Table Linkage disequilibrium results for 18 markers. (PDF)

S4 Table P-value of allelic richness between oases calculated by the Wilcoxon test. (PDF)

S5 Table P-value of expected heterozygosity between oases calculated by the Wilcoxon test. (PDF)

S6 Table P-value of observed heterozygosity calculated by the Wilcoxon test between oases. (PDF)

S7 Table P-value of Fis calculated by the Wilcoxon test between oases. (PDF)

S8 Table P-value of allelic richness between Structure populations calculated by the Wilcoxon test. (PDF)

S9 Table P-value of expected heterozygosity between Structure populations calculated by the Wilcoxon test. (PDF)

S10 Table P-value of observed heterozygosity between Structure populations calculated by the Wilcoxon test. (PDF)

S11 Table P-value of F_IS_ between Structure populations calculated by the Wilcoxon test. (PDF)

S12 Table Geographic distance (in km.) between oases. (PDF)
