# Supplementary figures and images for: Diversity of Algerian oases date palm (Phoenix dactylifera L., Arecaceae): Heterozygote excess and cryptic structure suggest farmer management had a major impact on diversity
Source: PLoS One. 2017 Apr 14;12(4):e0175232. doi: 10.1371/journal.pone.0175232 (PMC5391916; doi:10.1371/journal.pone.0175232)

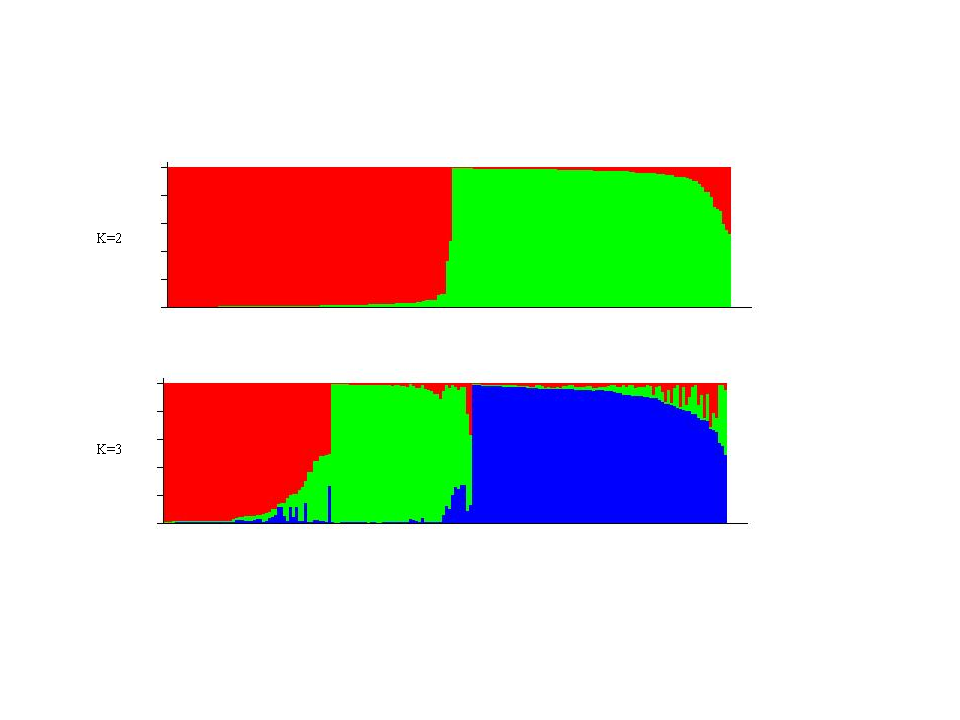

Supplement: S1 Fig — Individuals are represented by vertical colored lines. Individuals of the same color belong to the same cluster. Individuals with several different colors show the percentage of the genome that was inherited from each cluster. (TIF) [file pone.0175232.s001.tif]
