# Supplementary material for: Diversity of Algerian oases date palm (Phoenix dactylifera L., Arecaceae): Heterozygote excess and cryptic structure suggest farmer management had a major impact on diversity
Source: PLoS One. 2017 Apr 14;12(4):e0175232. doi: 10.1371/journal.pone.0175232 (PMC5391916; doi:10.1371/journal.pone.0175232)
Supplement: S1 Table — (PDF) [file pone.0175232.s002.pdf]

S1 Table. List of samples used in nuclear SSR genotyping.

Accession	Name of cultivar	Synonym	Oasis sampled	Latitude	Longitude	Origin /distribution
MS001	Deglet Nour		Biskra	34.85	5.73	Algeria, Tunisia
MS002	Deglet Nour		Biskra	34.85	5.73	Algeria, Tunisia
MS011	Ghars (Algerian)		Biskra	34.85	5.73	Algeria
MS021	Degla Baida		Biskra	34.85	5.73	Algeria
MS022	Degla Baida		Biskra	34.85	5.73	Algeria
MS031	Arichti		Biskra	34.85	5.73	Algeria, Tunisia
MS032	Arichti		Biskra	34.85	5.73	Algeria, Tunisia
MS041	Halwa		Biskra	34.85	5.73	Algeria
MS042	Litima		Biskra	34.85	5.73	Algeria
MS047	Dokhar (male)		Biskra	34.85	5.73	Algeria
MS048	Dokhar (male)		Biskra	34.85	5.73	Algeria
MS057	Mech Degla		Biskra	34.85	5.73	Algeria, Tunisia
MS058	Mech Degla		Biskra	34.85	5.73	Algeria, Tunisia
MS059	unknown		Biskra	34.85	5.73	Algeria
MS062	unknown		Biskra	34.85	5.73	Algeria
MS064	Halwaya		Biskra	34.85	5.73	Algeria
MS068	Zog El Moggar		Biskra	34.85	5.73	Algeria
MS070	Tanteboucht		Biskra	34.85	5.73	Algeria
MS071	Hamraya I		Biskra	34.85	5.73	Algeria
MS072	Tinicine		Biskra	34.85	5.73	Algeria
MS074	Tinicine		Touggourt	33.10	6.07	Algeria
MS077	Tanteboucht		Touggourt	33.10	6.07	Algeria
MS081	Tanteboucht	El Kayed	Touggourt	33.10	6.07	Algeria
MS082	Tanteboucht	El Kayed	Touggourt	33.10	6.07	Algeria
MS084	Tissibi		Touggourt	33.10	6.07	Algeria
MS085	Tissibi		Touggourt	33.10	6.07	Algeria
MS088	unknown		Touggourt	33.10	6.07	Algeria
MS089	Hamraya II		Touggourt	33.10	6.07	Algeria
MS090	Deglet Mech'a		Touggourt	33.10	6.07	Algeria
MS091	Tanslit		Touggourt	33.10	6.07	Algeria
MS092	Tifazwin		Touggourt	33.10	6.07	Algeria
MS094	Bouldjib		Touggourt	33.10	6.07	Algeria
MS095	Tazougart		Touggourt	33.10	6.07	Algeria
MS097	Taramount		Touggourt	33.10	6.07	Algeria
MS098	Takhoudrayt		Touggourt	33.10	6.07	Algeria
MS100	Tazougart		Touggourt	33.10	6.07	Algeria
MS101	Dimolo		Touggourt	33.10	6.07	Algeria
MS102	D'guel M'rass		Touggourt	33.10	6.07	Algeria
MS104	Takermoust		Touggourt	33.10	6.07	Algeria

MS105	Deglet Jito		Touggourt	33.10	6.07	Algeria
MS107	Tachlilat		Touggourt	33.10	6.07	Algeria
MS110	Chataya		Touggourt	33.10	6.07	Algeria
MS112	Deglet Nour (seed)		Touggourt	33.10	6.07	Algeria
MS113	Bayd Hmam		Touggourt	33.10	6.07	Algeria, Tunisia
MS115	D'guel El Hadj		Touggourt	33.10	6.07	Algeria
MS116	Tawadant		Touggourt	33.10	6.07	Algeria
MS118	Tawraghet		Touggourt	33.10	6.07	Algeria
MS119	Bahdid		Touggourt	33.10	6.07	Algeria
MS120	Kesba	Sokrya	Touggourt	33.10	6.07	Algeria
MS121	Bacheir		Touggourt	33.10	6.07	Algeria
MS122	Aghaliane		Touggourt	33.10	6.07	Algeria
MS124	Deglet Gurara		Touggourt	33.10	6.07	Algeria
MS127	Zaydi	Halimi	Oued Souf	33.37	6.87	Irak
MS128	Zaydi	Masri	Oued Souf	33.37	6.87	Irak
MS130	Halawi	Zohdi	Oued Souf	33.37	6.87	Irak
MS132	Tanteboucht		Oued Souf	33.37	6.87	Algeria
MS135	Abdel Azzaz		Oued Souf	33.37	6.87	Algeria
MS140	Dguel Sidi Khlil		Oued Souf	33.37	6.87	Algeria
MS141	Dguel Sidi Khlil		Oued Souf	33.37	6.87	Algeria
MS142	Amari		Oued Souf	33.37	6.87	Algeria, Tunisia
MS143	Amari		Oued Souf	33.37	6.87	Algeria, Tunisia
MS144	Alig (Algerian)		Oued Souf	33.37	6.87	Algeria
MS145	Alig (Algerian)		Oued Souf	33.37	6.87	Algeria
MS146	Hamraya		Oued Souf	33.37	6.87	Algeria
MS150	Tati		Oued Souf	33.37	6.87	Algeria
MS151	Tati		Oued Souf	33.37	6.87	Algeria
MS154	Halwa		Oued Souf	33.37	6.87	Algeria
MS156	Bukhannus		Oued Souf	33.37	6.87	Algeria
MS157	Bukhannus		Oued Souf	33.37	6.87	Algeria
MS158	Khadri		Oued Souf	33.37	6.87	Algeria
MS160	Dguel Mghas		Oued Souf	33.37	6.87	Algeria
MS162	Dfor Lgot		Oued Souf	33.37	6.87	Algeria
MS163	Dfor Lgot		Oued Souf	33.37	6.87	Algeria
MS164	Zaghraya		Oued Souf	33.37	6.87	Algeria
MS166	Horra		Oued Souf	33.37	6.87	Algeria, Tunisia
MS167	Horra		Oued Souf	33.37	6.87	Algeria, Tunisia
MS168	Alig (Algerian)	Bu'Rus	Oued Souf	33.37	6.87	Algeria
MS169	Alig (Algerian)	Bu'Rus	Oued Souf	33.37	6.87	Algeria
MS170	Tati		Oued Souf	33.37	6.87	Algeria
MS172	Lulu red		Oued Souf	33.37	6.87	Algeria
MS175	Lulu yellow		Oued Souf	33.37	6.87	Algeria
MS178	Kesba		Oued Souf	33.37	6.87	Algeria
MS179	Deglet Nour		Ouargla	31.96	5.35	Algeria, Tunisia

MS189	Ghars (Algerian)		Ouargla	31.96	5.35	Algeria
MS199	Tanslit		Ouargla	31.96	5.35	Algeria
MS205	Takermoust		Ouargla	31.96	5.35	Algeria
MS206	Tissibi		Ouargla	31.96	5.35	Algeria
MS208	Bent Qbala		Ghardaia	32.49	3.68	Algeria
MS209	Bent Qbala		Ghardaia	32.49	3.68	Algeria
MS218	Timjouhart		Ghardaia	32.49	3.68	Algeria
MS219	Timjouhart		Ghardaia	32.49	3.68	Algeria
MS224	Tafeziwin		Ghardaia	32.49	3.68	Algeria
MS225	Tafeziwin		Ghardaia	32.49	3.68	Algeria
MS229	Akerbouche		Ghardaia	32.49	3.68	Algeria
MS230	Akerbouche		Ghardaia	32.49	3.68	Algeria
MS231	Akerbouche		Ghardaia	32.49	3.68	Algeria
MS232	Akerbouche		Ghardaia	32.49	3.68	Algeria
MS233	Akerbouche		Ghardaia	32.49	3.68	Algeria
MS234	Akerbouche		Ghardaia	32.49	3.68	Algeria
MS238	Tissibi		Ghardaia	32.49	3.68	Algeria
MS242	Tazarzayet		Ghardaia	32.49	3.68	Algeria
MS243	Tazarzayet		Ghardaia	32.49	3.68	Algeria
MS245	Tamazouart		Ghardaia	32.49	3.68	Algeria
MS246	Ajoujil		Ghardaia	32.49	3.68	Algeria
MS247	Ajoujil		Ghardaia	32.49	3.68	Algeria
MS249	Tadmamt (Algerian)		Ghardaia	32.49	3.68	Algeria
MS253	Tadala		Ghardaia	32.49	3.68	Algeria
MS257	Tazizawt		Ghardaia	32.49	3.68	Algeria
MS258	Tazarzayet		Ghardaia	32.49	3.68	Algeria
MS260	Ououcht		Ghardaia	32.49	3.68	Algeria
MS262	Baydir		Ghardaia	32.49	3.68	Algeria
MS263	Baydir		Ghardaia	32.49	3.68	Algeria
MS266	Alig (Algerian)	Our'rous	Ghardaia	32.49	3.68	Algeria
MS270	Nasser Oussaleh		Ghardaia	32.49	3.68	Algeria
MS272	El Gachouche		Ghardaia	32.49	3.68	Algeria
MS273	El Gachouche		Ghardaia	32.49	3.68	Algeria
MS274	unknown		Tamanrasset	22.78	5.52	Algeria
MS275	unknown		Tamanrasset	22.78	5.52	Algeria
MS276	unknown		Tamanrasset	22.78	5.52	Algeria
MS277	unknown		Tamanrasset	22.78	5.52	Algeria
MS278	unknown		Tamanrasset	22.78	5.52	Algeria
MS279	unknown		Tamanrasset	22.78	5.52	Algeria
MS280	unknown		Tamanrasset	22.78	5.52	Algeria
MS281	Timjouhart		El Menia	30.59	2.88	Algeria
MS291	Deglet Nour		El Menia	30.59	2.88	Algeria, Tunisia
MS296	Ghars (Algerian)		El Menia	30.59	2.88	Algeria
MS301	Hmira		El Menia	30.59	2.88	Algeria

MS302	Hmira		El Menia	30.59	2.88	Algeria
MS307	unknown		El Menia	30.59	2.88	Algeria
MS316	Deglet Jdir		El Menia	30.59	2.88	Algeria
MS317	Deglet Jdir		El Menia	30.59	2.88	Algeria
MS326	Ouarglia		El Menia	30.59	2.88	Algeria
MS329	Ouarglia		El Menia	30.59	2.88	Algeria
MS331	Tinasser		El Menia	30.59	2.88	Algeria
MS332	Tinasser		El Menia	30.59	2.88	Algeria
MS336	Takerboucht		El Menia	30.59	2.88	Algeria
MS337	Takerboucht		El Menia	30.59	2.88	Algeria
MS338	Takerboucht		El Menia	30.59	2.88	Algeria
MS345	Tawragha		El Menia	30.59	2.88	Algeria
MS346	Tawragha		El Menia	30.59	2.88	Algeria
MS349	Ouzamig		Timimoun	29.25	0.23	Algeria
MS351	Chikh		Timimoun	29.25	0.23	Algeria
MS352	Adam Esof		Timimoun	29.25	0.23	Algeria
MS353	Adam Bulla		Timimoun	29.25	0.23	Algeria
MS356	Tgaza		Timimoun	29.25	0.23	Algeria
MS357	Azizaou	Adam Zrak	Timimoun	29.25	0.23	Algeria
MS358	Abbad		Timimoun	29.25	0.23	Algeria
MS360	Ighes N'wagada		Timimoun	29.25	0.23	Algeria
MS361	Gharas		Timimoun	29.25	0.23	Algeria
MS362	Tinrigh		Timimoun	29.25	0.23	Algeria
MS363	Azizaou	Adam Zrak	Timimoun	29.25	0.23	Algeria
MS365	Bukezzine		Timimoun	29.25	0.23	Algeria
MS366	Tinjdel		Timimoun	29.25	0.23	Algeria
MS367	Outaghsaït		Timimoun	29.25	0.23	Algeria
MS368	Timedjwel		Timimoun	29.25	0.23	Algeria
MS369	Tinazidhane		Timimoun	29.25	0.23	Algeria
MS372	Tinhud		Timimoun	29.25	0.23	Algeria
MS373	Aghammu		Adrar	27.87	-0.28	Algeria
MS374	Aghares		Adrar	27.87	-0.28	Algeria
MS375	Tinasser		Adrar	27.87	-0.28	Algeria
MS376	Deglet Wlad Mahmoud		Adrar	27.87	-0.28	Algeria
MS377	Chikh		Adrar	27.87	-0.28	Algeria
MS378	Banekhluf		Adrar	27.87	-0.28	Algeria
MS379	Mes'udiya		Adrar	27.87	-0.28	Algeria
MS380	Takerboucht		Adrar	27.87	-0.28	Algeria
MS381	Takerboucht		Adrar	27.87	-0.28	Algeria
MS382	Takerboucht		Adrar	27.87	-0.28	Algeria
MS383	Timakur		Adrar	27.87	-0.28	Algeria
MS384	Aharthan		Adrar	27.87	-0.28	Algeria
MS385	Asemmat		Adrar	27.87	-0.28	Algeria

MS388	Tilemsu		Adrar	27.87	-0.28	Algeria
MS389	Takerboucht		Adrar	27.87	-0.28	Algeria
MS390	Bent Cherk		Adrar	27.87	-0.28	Algeria
MS391	Bawa'adhim		Adrar	27.87	-0.28	Algeria
MS393	Fagous		Adrar	27.87	-0.28	Algeria, Morocco
MS394	Timliha		Adrar	27.87	-0.28	Algeria
MS395	Andekly		Adrar	27.87	-0.28	Algeria
MS396	Tindukan		Beni Abbes	30.13	-2.17	Algeria
MS397	Adam Bchir		Beni Abbes	30.13	-2.17	Algeria
MS398	Fagous		Beni Abbes	30.13	-2.17	Algeria, Morocco
MS399	Bent Cherk	Cherka	Beni Abbes	30.13	-2.17	Algeria
MS400	Adam Hror		Beni Abbes	30.13	-2.17	Algeria
MS401	Adam Tirnu		Beni Abbes	30.13	-2.17	Algeria
MS403	Harthan		Beni Abbes	30.13	-2.17	Algeria
MS404	Mcharret		Beni Abbes	30.13	-2.17	Algeria
MS405	Buféa		Beni Abbes	30.13	-2.17	Algeria
MS406	Kenta (Algerian)		Beni Abbes	30.13	-2.17	Algeria
MS407	Aharthan		Beni Abbes	30.13	-2.17	Algeria
MS408	Timrisa		Beni Abbes	30.13	-2.17	Algeria
MS410	Ma'tug		Beni Abbes	30.13	-2.17	Algeria
MS411	Chikh	Hamuri	Beni Abbes	30.13	-2.17	Algeria
MS413	Timliha		Beni Abbes	30.13	-2.17	Algeria

The name of the date palm cultivars analyzed with the geographical region and the latitude and longitude of the genotypes studied are presented.
