# Supplementary material for: Diversity of Algerian oases date palm (Phoenix dactylifera L., Arecaceae): Heterozygote excess and cryptic structure suggest farmer management had a major impact on diversity
Source: PLoS One. 2017 Apr 14;12(4):e0175232. doi: 10.1371/journal.pone.0175232 (PMC5391916; doi:10.1371/journal.pone.0175232)
Supplement: S2 Table — (PDF) [file pone.0175232.s003.pdf]

S2 Table. Genetic assignment of the sample cultivars according to chlorotype and STRUCTURE grouping for K = 3.

**CV name**	**N° of samples***	**Chlorotype**	**Ancestry G1**	**Ancestry G2**	**Ancestry G3**
Abbad	1/1	occidental	06.7	93.3	00.0
Abdel Azzaz	1/5	oriental	100.0	00.0	00.0
Adam Bchir	1/1	oriental	01.7	05.0	93.3
Adam Bulla	1/1	oriental	03.3	06.7	90.0
Adam Esof	1/1	oriental	03.3	06.7	90.0
Adam Hror	1/1	oriental	06.7	06.7	86.6
Adam Tirnu	1/1	oriental	01.7	03.3	95.0
Aghaliane	1/2	oriental	51.7	48.3	00.0
Aghammu	1/1	oriental	03.3	16.7	80.0
Aghares	1/1	occidental	01.7	03.3	95.0
Aharthan	2/2	oriental	01.0	00.0	99.0
Ajoujil	2/3	oriental	0.66	03.0	96.4
Akerbouche	6/8	oriental	100	00.0	00.0
Alig (Algerian)	5/8	occidental	01.0	99.0	00.0
Amari	2/2	occidental	08.3	91.7	00.0
Andekly	1/1	oriental	01.6	06.7	91.7
Arichti	2/10	occidental	56.7	43.3	00.0
Asemmat	1/1	oriental	01.6	41.7	56.7
Azizaou	2/2	oriental	03.3	00.0	96.7
Bacheir	1/1	oriental	96.7	01.6	01.7
Bahdid	1/1	oriental	96.7	03.3	00.0
Banekhluf	1/1	oriental	01.7	08.3	90.0
Bawa'adhim	1/1	oriental	01.7	00.0	98.3
Bayd Hmam	1/2	occidental	53.3	46.7	00.0
Baydir	2/4	occidental	01.7	98.3	00.0
Bent Cherk	2/2	occidental	03.3	01.7	95.0
Bent Qbala	2/11	oriental	01.7	01.6	96.7
Bouldjib	1/1	oriental	30.0	01.7	68.3

Buféa	1/1	occidental	01.7	15.0	83.3
Bukezzine	1/1	oriental	01.7	03.3	95.0
Bukhannus	2/2	occidental	05.0	95.0	00.0
Chataya	1/2	oriental	80.0	15.0	05.0
Chikh	3/3	occidental	03.3	70.0	26.7
Degla Baida	3/19	occidental	01.0	99.0	00.0
Deglet Gurara	1/3	occidental	23.3	68.4	08.3
Deglet Jdir	2/10	occidental	01.7	01.6	96.7
Deglet Jito	1/2	oriental	36.7	50.0	13.3
Deglet Mech'a	1/1	oriental	94.0	05.0	01.0
Deglet Nour	4/25	oriental	01.0	99.0	00.0
Deglet Wlad Mahmoud	1/1	occidental	01.7	08.3	90.0
Dfor Lgot	2/2	oriental	99.3	00.7	00.0
Dguel Mghas	1/2	occidental	70.0	15.0	15.0
Dguel Sidi Khlil	2/2	oriental	93.3	01.6	01.7
Dimolo	1/1	oriental	93.3	06.7	00.0
D'guel El Hadj	1/1	oriental	63.3	36.7	00.0
D'guel M'rass	1/1	oriental	76.7	23.3	00.0
El Gachouche	2/2	oriental	95.0	05.0	00.0
Fagous	1/2	occidental	01.7	76.7	21.6
Gharas	1/2	oriental	03.3	00.0	96.7
Ghars (Algerian)	3/25	oriental	99.0	00.0	01.0
Halawi	1/2	oriental	16.7	03.3	80.0
Halwa	2/3	occidental	00.0	100.0	00.0
Halwaya	1/4	occidental	00.0	100.0	00.0
Hamraya I	1/1	oriental	73.3	26.7	00.0
Hamraya II	1/1	oriental	100.0	00.0	00.0
Hamraya	1/4	occidental	50.0	23.3	26.7
Harthan	1/1	occidental	01.0	06.7	92.3
Hmira	2/7	oriental	01.7	00.0	98.3
Horra	2/2	oriental	00.0	100.0	00.0
Ighes N'wagada	1/1	oriental	00.7	09.3	90.0

Kenta (Algerian)	1/1	occidental	01.7	05.0	93.3
Kesba	2/2	oriental	86.7	03.3	11.7
Khadri	1/2	oriental	91.7	05.0	03.3
Litima	1/1	oriental	00.7	94.0	05.3
Lulu red	1/3	oriental	83.3	16.7	00.0
Lulu yellow	1/3	oriental	90.0	05.0	05.0
Ma'tug	1/1	oriental	25.0	01.7	73.3
Mcharret	1/1	oriental	03.3	01.0	95.7
Mech Degla	2/7	occidental	01.0	99.0	00.0
Mes'udiya	1/1	occidental	20.0	13.3	66.7
Nasser Oussaleh	1/2	oriental	06.7	43.3	50.0
Ouarglia	2/5	occidental	03.3	96.7	00.0
Ououcht	1/2	oriental	03.3	10.0	86.7
Outaghsaït	1/1	occidental	06.7	10.0	83.3
Ouzamig	1/1	oriental	01.0	00.0	99.0
Tachlilat	1/3	oriental	95.0	03.3	01.7
Tadala	1/4	oriental	16.7	03.3	80.0
Tadmamt (Algerian)	1/5	occidental	13.3	86.7	00.0
Tafeziwin	2/5	oriental	01.7	01.6	96.7
Takerboucht	7/14	occidental	03.3	01.7	95.0
Takermoust	2/3	oriental	93.4	03.3	03.3
Takhoudrayt	1/2	occidental	06.7	86.7	06.6
Tamazouart	1/1	occidental	05.0	95.0	00.0
Tanslit	2/7	oriental	15.7	01.0	83.3
Tanteboucht	5/9	oriental	100.0	00.0	00.0
Taramount	1/1	oriental	63.3	36.7	00.0
Tati	3/4	oriental	100.0	00.0	00.0
Tawadant	1/2	oriental	53.3	46.7	00.0
Tawragha	2/5	oriental	01.7	01.6	96.7
Tawraghet	1/1	oriental	78.3	10.0	11.7
Tazarzayet	3/5	oriental	00.0	00.0	100.0
Tazizawt	1/3	occidental	03.3	20.0	76.7

Tazougart	2/2	oriental	93.3	06.7	00.0
Tgaza	1/	oriental	-	-	-
Tifazwin	1/2	oriental	26.7	10.0	63.3
Tilemsu	1/1	oriental	03.3	03.3	93.4
Timakur	1/1	occidental	01.7	96.7	01.6
Timedjwel	1/1	occidental	10.0	16.7	73.3
Timjouhart	3/6	oriental	01.0	00.0	99.0
Timliha	2/3	oriental	00.7	01.0	98.3
Timrisa	1/1	oriental	05.0	95.0	00.0
Tinasser	3/7	occidental	03.3	06.7	90.0
Tinazidhane	1/1	occidental	01.0	43.3	55.7
Tindukan	1/1	occidental	03.3	85.0	11.7
Tinhud	1/1	oriental	00.7	20.0	79.3
Tinicine	2/4	oriental	100.0	00.0	00.0
Tinjdel	1/1	occidental	10.0	10.0	80.0
Tinrigh	1/1	oriental	03.3	06.7	90.0
Tissibi	4/9	oriental	03.3	00.0	98.7
Zaghraya	1/2	oriental	16.7	10.0	73.3
Zaydi	2/3	oriental	03.3	05.0	91.7
Zog El Moggar	1/2	oriental	85.0	10.0	05.0

*First data ABI genotyping of nuclear and chloroplast SSR, second data Qiaxcel genotyping of chloroplast minisatellites
