# Supplementary material for: Diversity of Algerian oases date palm (Phoenix dactylifera L., Arecaceae): Heterozygote excess and cryptic structure suggest farmer management had a major impact on diversity
Source: PLoS One. 2017 Apr 14;12(4):e0175232. doi: 10.1371/journal.pone.0175232 (PMC5391916; doi:10.1371/journal.pone.0175232)
Supplement: S3 Table — (PDF) [file pone.0175232.s004.pdf]

S3 Table. Linkage disequilibrium results for 18 markers.

	loc1	loc2	r2
1	mPdIRD031	mPdIRD033	0.000382717269438089
2	mPdIRD031	mPdIRD040	0.000822388876452006
3	mPdIRD031	PdCUC3_ssr2	0.0271795508479885
4	mPdIRD031	PdAP3_ssr_F4	0.000649587886271771
5	mPdIRD031	mPdCIR078	0.000196879685691566
6	mPdIRD031	mPdCIR015	0.00354594048629652
7	mPdIRD031	mPdCIR016	0.00435305063161379
8	mPdIRD031	mPdCIR032	0.0686420404739059
9	mPdIRD031	mPdCIR035	0.000181738826494775
10	mPdIRD031	mPdCIR057	0.0402700890143889
11	mPdIRD031	mPdCIR085	0.0117550249019509
12	mPdIRD031	PdAG1_ssr	0.00627085409601604
13	mPdIRD031	mPdCIR010	0.00865406596572999
14	mPdIRD031	mPdCIR025	0.0100155045173372
15	mPdIRD031	mPdCIR063	2.99714210511709e-06
16	mPdIRD031	PdCUC3_ssr1	0
17	mPdIRD031	mPdIRD013	0.00990365788133385
18	mPdIRD033	mPdIRD040	0.000843644544431946
19	mPdIRD033	PdCUC3_ssr2	3.06683815971309e-06
20	mPdIRD033	PdAP3_ssr_F4	0.00584623997585936
21	mPdIRD033	mPdCIR078	0.000216711229324077
22	mPdIRD033	mPdCIR015	0.00233237065236185
23	mPdIRD033	mPdCIR016	0.0343991294727815
24	mPdIRD033	mPdCIR032	0.0102293113101658
25	mPdIRD033	mPdCIR035	0.0255571918678574
26	mPdIRD033	mPdCIR057	0.000216581146283063
27	mPdIRD033	mPdCIR085	0.00757045925036749
28	mPdIRD033	PdAG1_ssr	0.0153438440752749
29	mPdIRD033	mPdCIR010	0.00293977149699714
30	mPdIRD033	mPdCIR025	0.00894121385924665
31	mPdIRD033	mPdCIR063	0.00141873290422829
32	mPdIRD033	PdCUC3_ssr1	0
33	mPdIRD033	mPdIRD013	0.00115635704975388
34	mPdIRD040	PdCUC3_ssr2	0.0435248620910081
35	mPdIRD040	PdAP3_ssr_F4	0.000403065384481567
36	mPdIRD040	mPdCIR078	0.011015234113419
37	mPdIRD040	mPdCIR015	0.00268197237008597
38	mPdIRD040	mPdCIR016	0.011526992356459
39	mPdIRD040	mPdCIR032	0.00254219346770406
40	mPdIRD040	mPdCIR035	0.00249918523283886

41	mPdIRD040	mPdCIR057	0.00918871463574244
42	mPdIRD040	mPdCIR085	0.000516988472570942
43	mPdIRD040	PdAG1_ssr	0.0311696794492949
44	mPdIRD040	mPdCIR010	0.0467214366507295
45	mPdIRD040	mPdCIR025	0.010526814050473
46	mPdIRD040	mPdCIR063	0.0466379677354182
47	mPdIRD040	PdCUC3_ssr1	0
48	mPdIRD040	mPdIRD013	0.0192680424124957
49	PdCUC3_ssr2	PdAP3_ssr_F4	0.00030016447764237
50	PdCUC3_ssr2	mPdCIR078	0.00992944811978705
51	PdCUC3_ssr2	mPdCIR015	4.15350934417593e-07
52	PdCUC3_ssr2	mPdCIR016	0.0235915181063782
53	PdCUC3_ssr2	mPdCIR032	0.0670358531957313
54	PdCUC3_ssr2	mPdCIR035	0.00192334753781248
55	PdCUC3_ssr2	mPdCIR057	0.000393077224223495
56	PdCUC3_ssr2	mPdCIR085	0.0118622175417075
57	PdCUC3_ssr2	PdAG1_ssr	0.000241370997203613
58	PdCUC3_ssr2	mPdCIR010	0.0273619660351858
59	PdCUC3_ssr2	mPdCIR025	0.0109160087438354
60	PdCUC3_ssr2	mPdCIR063	0.0106657183395729
61	PdCUC3_ssr2	PdCUC3_ssr1	0
62	PdCUC3_ssr2	mPdIRD013	0.00197914801612925
63	PdAP3_ssr_F4	mPdCIR078	0.000353416952938039
64	PdAP3_ssr_F4	mPdCIR015	0.012868985144043
65	PdAP3_ssr_F4	mPdCIR016	0.0372238328421604
66	PdAP3_ssr_F4	mPdCIR032	0.00366220145939701
67	PdAP3_ssr_F4	mPdCIR035	0.0327862259673023
68	PdAP3_ssr_F4	mPdCIR057	0.0382637570740716
69	PdAP3_ssr_F4	mPdCIR085	0.0040597506840609
70	PdAP3_ssr_F4	PdAG1_ssr	0.0138334991148758
71	PdAP3_ssr_F4	mPdCIR010	0.0202039758803603
72	PdAP3_ssr_F4	mPdCIR025	0.000944403052736939
73	PdAP3_ssr_F4	mPdCIR063	0.00696588071637712
74	PdAP3_ssr_F4	PdCUC3_ssr1	0
75	PdAP3_ssr_F4	mPdIRD013	0.00017387752182595
76	mPdCIR078	mPdCIR015	0.157724289513712
77	mPdCIR078	mPdCIR016	0.00072929331010063
78	mPdCIR078	mPdCIR032	0.00517569841564749
79	mPdCIR078	mPdCIR035	0.0106390655888397
80	mPdCIR078	mPdCIR057	0.0362462955919366
81	mPdCIR078	mPdCIR085	0.0464112771768544
82	mPdCIR078	PdAG1_ssr	0.0102459907840628
83	mPdCIR078	mPdCIR010	0.0512471812614954
84	mPdCIR078	mPdCIR025	0.0560281233385465
85	mPdCIR078	mPdCIR063	0.0128823849747096

86	mPdCIR078	PdCUC3_ssr1	0
87	mPdCIR078	mPdIRD013	0.000744843698296168
88	mPdCIR015	mPdCIR016	0.00655701532439451
89	mPdCIR015	mPdCIR032	0.0447549248719361
90	mPdCIR015	mPdCIR035	0.000624867549370315
91	mPdCIR015	mPdCIR057	0.0404302402188928
92	mPdCIR015	mPdCIR085	0.00612864323465014
93	mPdCIR015	PdAG1_ssr	0.00636494491910748
94	mPdCIR015	mPdCIR010	0.0355834138025111
95	mPdCIR015	mPdCIR025	0.0905960203476982
96	mPdCIR015	mPdCIR063	0.0340767947492435
97	mPdCIR015	PdCUC3_ssr1	0
98	mPdCIR015	mPdIRD013	0.0211152271082638
99	mPdCIR016	mPdCIR032	0.0118907476415877
100	mPdCIR016	mPdCIR035	0.0411819886423851
101	mPdCIR016	mPdCIR057	0.00972361861470236
102	mPdCIR016	mPdCIR085	0.000698231943996028
103	mPdCIR016	PdAG1_ssr	0.00369878937633725
104	mPdCIR016	mPdCIR010	0.00596824398785828
105	mPdCIR016	mPdCIR025	0.112522498167946
106	mPdCIR016	mPdCIR063	4.29213978634287e-05
107	mPdCIR016	PdCUC3_ssr1	0
108	mPdCIR016	mPdIRD013	6.37577093696913e-05
109	mPdCIR032	mPdCIR035	5.9466931961294e-05
110	mPdCIR032	mPdCIR057	0.0167377165727428
111	mPdCIR032	mPdCIR085	0.000878357866051523
112	mPdCIR032	PdAG1_ssr	0.00442372272925404
113	mPdCIR032	mPdCIR010	0.01973657148106
114	mPdCIR032	mPdCIR025	0.0121918852818
115	mPdCIR032	mPdCIR063	0.00916681777934644
116	mPdCIR032	PdCUC3_ssr1	0
117	mPdCIR032	mPdIRD013	0.00237260182553454
118	mPdCIR035	mPdCIR057	0.0682931543791107
119	mPdCIR035	mPdCIR085	0.0146866909790032
120	mPdCIR035	PdAG1_ssr	0.00371496081308918
121	mPdCIR035	mPdCIR010	0.0159795591517684
122	mPdCIR035	mPdCIR025	0.00687452935415063
123	mPdCIR035	mPdCIR063	1.71184419650046e-05
124	mPdCIR035	PdCUC3_ssr1	0
125	mPdCIR035	mPdIRD013	0.0107932495818951
126	mPdCIR057	mPdCIR085	0.0191295102702965
127	mPdCIR057	PdAG1_ssr	0.00364340394894195
128	mPdCIR057	mPdCIR010	0.000516585409228642
129	mPdCIR057	mPdCIR025	0.000319631533350089
130	mPdCIR057	mPdCIR063	0.000764036872971146

131	mPdCIR057	PdCUC3_ssr1	0
132	mPdCIR057	mPdIRD013	0.000870814710220407
133	mPdCIR085	PdAG1_ssr	0.00012700155375995
134	mPdCIR085	mPdCIR010	0.000588382595782908
135	mPdCIR085	mPdCIR025	0.010525856088996
136	mPdCIR085	mPdCIR063	0.000141900076560041
137	mPdCIR085	PdCUC3_ssr1	0
138	mPdCIR085	mPdIRD013	0.0076380458861821
139	PdAG1_ssr	mPdCIR010	0.0239631488581693
140	PdAG1_ssr	mPdCIR025	0.054852635725666
141	PdAG1_ssr	mPdCIR063	0.00735688774245966
142	PdAG1_ssr	PdCUC3_ssr1	0
143	PdAG1_ssr	mPdIRD013	0.0225096562443351
144	mPdCIR010	mPdCIR025	0.000987657148325623
145	mPdCIR010	mPdCIR063	0.00459492875906347
146	mPdCIR010	PdCUC3_ssr1	0
147	mPdCIR010	mPdIRD013	0.00599525735464234
148	mPdCIR025	mPdCIR063	0.00461608664522858
149	mPdCIR025	PdCUC3_ssr1	0
150	mPdCIR025	mPdIRD013	0.0227130562326093
151	mPdCIR063	PdCUC3_ssr1	0
152	mPdCIR063	mPdIRD013	0.0389128572687169
153	PdCUC3_ssr1	mPdIRD013	0
