# Supplementary material for: Diversity of Algerian oases date palm (Phoenix dactylifera L., Arecaceae): Heterozygote excess and cryptic structure suggest farmer management had a major impact on diversity
Source: PLoS One. 2017 Apr 14;12(4):e0175232. doi: 10.1371/journal.pone.0175232 (PMC5391916; doi:10.1371/journal.pone.0175232)
Supplement: S4 Table — (PDF) [file pone.0175232.s005.pdf]

S4 Table. P-value of allelic richness between oases calculated by the Wilcoxon test.

	Biskra	Touggourt	Oued Souf	Ouargla	Ghardaia	Tamanrasset	El Menia	Timimoun	Adrar	Beni Abbes
Biskra		0.8176	0.05688	0.1901	0.6777	0.2247	0.8176	0.3289	0.6112	0.05054
Touggourt			0.1148	0.1055	0.8176	0.2891	0.816	0.306	0.5791	0.06483
Oued Souf				0.6603	0.1743	0.5872	0.1272	0.8176	0.05688	0.1594
Ouargla					0.2435	0.3935	0.1272	0.463	0.2633	1.0000
Ghardaia						0.4874	0.9632	0.4874	0.9265	0.05224
Tamanrasset							0.518	1.0000	0.4874	0.3318
El Menia								0.306	0.89	0.07968
Timimoun									0.2247	0.1202
Adrar										0.002487
Beni Abbes										

The bonferroni corrected p-value for 40 tests is 0.00125.
