# Supplementary material for: Diversity of Algerian oases date palm (Phoenix dactylifera L., Arecaceae): Heterozygote excess and cryptic structure suggest farmer management had a major impact on diversity
Source: PLoS One. 2017 Apr 14;12(4):e0175232. doi: 10.1371/journal.pone.0175232 (PMC5391916; doi:10.1371/journal.pone.0175232)
Supplement: S5 Table — (PDF) [file pone.0175232.s006.pdf]

S5 Table. P-value of expected heterozygosity between oases calculated by the Wilcoxon test.

	Biskra	Touggourt	Oued Souf	Ouargla	Ghardaia	Tamanrasset	El Menia	Timimoun	Adrar	Beni Abbes
Biskra		1.0000	0.2583	0.2659	0.9773	0.7332	0.7041	0.485	0.6599	0.1472
Touggourt			0.1727	0.4953	0.5698	0.8506	0.6492	0.3384	0.6359	0.2242
Oued Souf				0.1052	0.191	0.3485	0.205	0.776	0.2761	0.5505
Ouargla					0.4344	0.5756	0.9095	0.1126	0.32	0.06826
Ghardaia						0.7367	0.7536	0.9176	0.7982	0.32
Tamanrasset							0.7959	0.6191	0.5681	0.118
El Menia								0.4923	0.3277	0.1928
Timimoun									0.6699	0.2452
Adrar										0.1084
Beni Abbes										

The bonferroni corrected p-value for 40 tests is 0.00125.
