# Supplementary material for: Diversity of Algerian oases date palm (Phoenix dactylifera L., Arecaceae): Heterozygote excess and cryptic structure suggest farmer management had a major impact on diversity
Source: PLoS One. 2017 Apr 14;12(4):e0175232. doi: 10.1371/journal.pone.0175232 (PMC5391916; doi:10.1371/journal.pone.0175232)
Supplement: S6 Table — (PDF) [file pone.0175232.s007.pdf]

S6 Table. P-value of observed heterozygosity calculated by the Wilcoxon test between oases.

	Biskra	Touggourt	Oued Souf	Ouargla	Ghardaia	Tamanrasset	El Menia	Timimoun	Adrar	Beni Abbes
Biskra		1.0000	0.6191	0.609	0.2247	0.7946	0.8766	0.2093	0.2093	0.8313
Touggourt			0.623	0.5889	0.09272	0.776	0.8361	0.2866	0.67	0.7225
Oued Souf				0.8502	0.4262	0.698	0.5348	0.4776	0.5699	0.6777
Ouargla					0.5694	0.3788	0.5096	0.6699	0.3379	0.6358
Ghardaia						0.2891	0.3519	0.4629	0.2272	0.1849
Tamanrasset							0.8202	0.2558	1.0000	1.0000
El Menia								0.5224	0.8313	0.887
Timimoun									0.1268	0.3678
Adrar										0.9246
Beni Abbes										

The bonferroni corrected p-value for 40 tests is 0.00125.
