# Supplementary material for: Diversity of Algerian oases date palm (Phoenix dactylifera L., Arecaceae): Heterozygote excess and cryptic structure suggest farmer management had a major impact on diversity
Source: PLoS One. 2017 Apr 14;12(4):e0175232. doi: 10.1371/journal.pone.0175232 (PMC5391916; doi:10.1371/journal.pone.0175232)
Supplement: S7 Table — (PDF) [file pone.0175232.s008.pdf]

S7 Table. P-value of Fis calculated by the Wilcoxon test between oases.

	Biskra	Touggourt	Oued Souf	Ouargla	Ghardaia	Tamanrasset	El Menia	Timimoun	Adrar	Beni Abbes
Biskra		0.293	0.5871	0.6257	0.03615	0.2522	0.7546	0.89	0.7761	0.552
Touggourt			0.2453	0.7609	0.1639	0.1727	0.8339	0.804	0.2933	0.2524
Oued Souf				0.4631	0.01244	0.3487	0.6981	0.7563	0.9799	0.4851
Ouargla					0.391	0.3258	0.7775	0.9032	0.583	0.5097
Ghardaia						0.0437	0.3654	0.05249	0.0883	0.01126
Tamanrasset							0.155	0.2681	0.3935	0.7775
El Menia								0.8564	0.6494	0.485
Timimoun									0.4924	0.3464
Adrar										0.6233
Beni Abbes										
