# Supplementary material for: Diversity of Algerian oases date palm (Phoenix dactylifera L., Arecaceae): Heterozygote excess and cryptic structure suggest farmer management had a major impact on diversity
Source: PLoS One. 2017 Apr 14;12(4):e0175232. doi: 10.1371/journal.pone.0175232 (PMC5391916; doi:10.1371/journal.pone.0175232)
Supplement: S10 Table — (PDF) [file pone.0175232.s011.pdf]

S10 Table. P-value of observed heterozygosity between Structure populations calculated by the Wilcoxon test.

	population 1	population 2	population 3
population 1		0.4486	1.0000
population 2			0.2442
population 3			
