# Supplementary material for: Diversity of Algerian oases date palm (Phoenix dactylifera L., Arecaceae): Heterozygote excess and cryptic structure suggest farmer management had a major impact on diversity
Source: PLoS One. 2017 Apr 14;12(4):e0175232. doi: 10.1371/journal.pone.0175232 (PMC5391916; doi:10.1371/journal.pone.0175232)
Supplement: S11 Table — (PDF) [file pone.0175232.s012.pdf]

S11 Table. P-value of F_{IS} between Structure populations calculated by the Wilcoxon test.

	population 1	population 2	population 3
population 1		0.03182	0.2242
population 2			0.2242
population 3			
