# Supplementary material for: Diversity of Algerian oases date palm (Phoenix dactylifera L., Arecaceae): Heterozygote excess and cryptic structure suggest farmer management had a major impact on diversity
Source: PLoS One. 2017 Apr 14;12(4):e0175232. doi: 10.1371/journal.pone.0175232 (PMC5391916; doi:10.1371/journal.pone.0175232)
Supplement: S12 Table — (PDF) [file pone.0175232.s013.pdf]

**S12 Table.** Geographic distance (in km.) between oases.

| | Biskra | Touggourt | Oued Souf | Ouargla | Ghardaia | Tamanrasset | El Menia | Timimoun | Adrar | Beni Abbes |
|---|---|---|---|---|---|---|---|---|---|---|
| Biskra | | 197.1 | 195.184 | 323.283 | 323.792 | 1342.278 | 543.499 | 809.887 | 962.865 | 907.483 |
| Touggourt | | | 80.234 | 143.612 | 233.462 | 1148.796 | 410.673 | 701.176 | 841.323 | 846.935 |
| Oued Souf | | | | 211.716 | 313.373 | 1184.941 | 486.936 | 779.304 | 917.189 | 927.145 |
| Ouargla | | | | | 167.779 | 1020.907 | 279.825 | 575.136 | 707.785 | 744.558 |
| Ghardaia | | | | | | 1094.746 | 224.459 | 488.006 | 639.239 | 614.465 |
| Tamanrasset | | | | | | | 907.072 | 892.454 | 812.244 | 1119.124 |
| El Menia | | | | | | | | 295.667 | 430.651 | 487.179 |
| Timimoun | | | | | | | | | 161.33 | 251.629 |
| Adrar | | | | | | | | | | 311.333 |
| Beni Abbes | | | | | | | | | | |
